# Comparing the effect of zinc oxide and titanium dioxide nanoparticles on the ability of moderately halophilic bacteria to treat wastewater

**DOI:** 10.1038/s41598-021-96413-5

**Published:** 2021-08-20

**Authors:** Vanessa Weber, Ilunga Kamika, Maggy N. B. Momba

**Affiliations:** 1 Department of Environmental, Water and Earth Sciences, Tshwane University of Technology, Arcadia Campus, Private Bag X680, Pretoria, 0001 South Africa; 2 Institute for Nanotechnology and Water Sustainability, School of Science, College of Science, Engineering and Technology, University of South Africa, Florida Campus, Johannesburg, South Africa

**Keywords:** Biotechnology, Environmental biotechnology

## Abstract

This study evaluates the ability of moderately halophilic bacterial isolates (*Serratia* sp.*, Bacillus* sp.*, Morganella* sp.*, Citrobacter freundii and Lysinibacillus* sp.) to treat polluted wastewater in the presence of nZnO and nTiO_2_ nanoparticles. In this study, bacteria isolates were able to take up nZnO and nTiO_2_ at concentrations ranging from 1 to 50 mg/L in the presence of higher DO uptake at up to 100% and 99%, respectively, while higher concentrations triggered a significant decrease. Individual halophilic bacteria exhibited a low COD removal efficiency in the presence of both metal oxide nanoparticles concentration ranged between 1 and 10 mg/L. At higher concentrations, they triggered COD release of up to − 60% concentration. Lastly, the test isolates also demonstrated significant nutrient removal efficiency in the following ranges: 23–65% for NO_3_^−^ and 28–78% for PO_4_^3−^. This study suggests that moderately halophilic bacteria are good candidates for the bioremediation of highly polluted wastewater containing low metal oxide nanoparticles.

## Introduction

Industrialisation and rapid urbanisation have resulted in the pollution of water resources, worldwide, at an alarming rate^1^. In South Africa, the average rainfall of 450 mm per annum is far below the global average of 860 mm per year and the country is characterised as a water-scarce country^2^. Water-stressed countries are more vulnerable to changes in water availability such as polluted water sources. Industrial wastewaters, in particular, are a huge source of water pollution as they contain very high concentrations of several pollutants of public health concern when disposed of in the receiving water bodies^2,3^. Nanoparticles are also used in the production of many consumer products including sunscreens, dye-sensitised solar cells, paints, textiles, electronics, pharmaceuticals, drug delivery, clothing, tyres, sporting goods, as well as in diagnostics, for medical purposes and surgery^4,5^. Although they have beneficial properties for product development, those same properties present uncertainty as there may be potential environmental and health risks in regard to the product development, use and end-of-life. Several non-governmental organisations and associations have been calling for increased research efforts to uncover the health and environmental risks of nanomaterials, while many NMs are still in the early stages of development^6^. Studies of water quality in various effluents revealed that anthropogenic activities have an important negative impact on water quality^7,8^. This is a result of inadequate wastewater treatment, which is further characterized by important modifications of pH, DO, COD, nitrate, phosphate content, and so forth^9^. Hence, a cost-effective treatment process is crucial to prevent further risks of environmental pollution, which may lead to human health problems. Several techniques have been developed in the past for the detoxification of industrial effluents such as ion exchange, reverse osmosis, chemical precipitation, coagulation, electrolysis processes and adsorption^10^. However, due to the fact that physicochemical treatments have shown several disadvantages, biological treatment has become the preferred option because of the ability of microorganisms to significantly reduce the quantity of pollutants in aqueous solutions^11–14^. Even though a number of microorganisms are known for their abilities to decontaminate wastewater^15^, very few studies on the capability of the halophilic bacteria to degrade pollutants in wastewater have been conducted^11^. In their study, Woolard and Irvine^16^ reported that halophilic microorganisms are suitable to remove organic matter without dilution in a biological degradation process. Not only can these microorganisms survive and grow under extreme conditions, but usually, they require these conditions for survival and growth^17^. Of these microorganisms, moderate halophiles thrive in habitats with moderate salt concentrations (3–15% NaCl), but can also grow under extreme conditions^18^. The present study, therefore, aimed at evaluating the ability of selected moderately halophilic bacterial isolates to treat polluted wastewater in the presence of nZnO and nTiO_2_ nanoparticles. This study was conducted in laboratory-scale reactors.

## Materials and methods

### Test organisms

The moderately halophilic bacteria were isolated from brine samples collected at the Emalahleni Water Reclamation Plant*.* The halophilic bacteria isolated were *Serratia* sp.*, Bacillus* sp.*, Morganella* sp.*, Citrobacter freundii* and *Lysinibacillus* sp. Isolation and enrichment of halophilic bacteria were performed using halophiles moderate (HM) medium with and without agar. The HM with agar was supplemented with 98 g NaCl, 2 g KCl, 1 g MgSO_4_·7H_2_O, 0.36 g CaCl_2_·2H_2_O, 0.06 g NaHCO_3_, 0.24 g NaBr, 1 g FeCl_3_·6H_2_O, 10 g bacto tryptone (Difco), 1 g glucose and 20 g agar per 100 mL^19^. The media were autoclaved (121 °C for 15 min) and thereafter incubated overnight at 37 °C. Only media, which showed no growth were used. To isolate halophilic bacteria, 1 mL of the brine sample was added to an Erlenmeyer flask containing 50 mL of HM liquid medium. After incubation in an orbital shaker (shaking speed of 100 rpm) at 37 °C for 30 min, 1 mL of supernatant was added to 9 mL of sterile saline water (0.85%) for serial dilutions and plated on a Halobacillus medium (HbM) agar at pH 7.5 and later incubated at 37 °C overnight. Phenotypic characterisation of each isolate was done based on morphological, physiological and biochemical tests.

### Sample collection and preparation of modified wastewater mixed liquor

The municipal wastewater samples were collected between February and April 2017 from the primary settling tank of the Daspoort Wastewater Treatment Plant (a domestic wastewater plant situated in the centre of Pretoria/South Africa). Prior to filtration, the samples were allowed to settle for 2 h, then filtered using Whatman No. 1 filter papers (Sigma Aldrich, SA) to remove biomass and other suspended solids. The filtered sample profile was determined in terms of the chemical oxygen demand (COD) using closed reflux methods according to the standard methods (APHA, 2001), dissolved oxygen (DO) and pH using a pH probe (Model: PHC101, HACH) and DO probe (Model: LDO, HACH). The Zn and Ti concentrations were determined from pre-acidified samples using the inductively-coupled plasma optical emission spectrometer (ICP-OES) (AMETEK-Spectro Analytical Instruments GmbH & Co., Kleve, Germany).

To prepare the modified wastewater mixed liquor media, wastewater samples with concentrations of Zn and Ti less than 1 mg/L were used. The chemicals such as C_12_H_22_O_11_ (20 mg/L, sucrose), NaCl (40 mg/L), KNO_3_ (0.18 g/L) and MgSO_4_·7H_2_O (0.5 g/L), used as nutrient and carbon sources for the growth of the moderately halophilic bacteria, were considered^20^.

### Characterisation of commercial ZnO and TiO_2_ nanoparticles

Prior to use, commercial ZnO and TiO_2_ nanoparticles with < 100 nm particle size purchased from Sigma-Aldrich (South Africa) were characterised in terms of morphology, particle size and chemical composition using a high-resolution transmission electron microscope (TEM, JEOL-JEM 2100), which operates at 200 kV. The TEM analysis involved the fixation-penetration of nanoparticles; the fixative was allowed to slowly trickle down the side of the tube, whereupon the samples were dried at room temperature before the TEM examination. The TEM analysis was performed with a scanning TEM (STEM) system for dark-field and bright-field analysis, selected area diffraction analysis and equipped with an Oxford Inca energy-dispersive silicon-drift X-ray (EDX) spectrometer for compositional analysis. Image acquisition was performed with a Gatan Orius™ bottom mount, 14-bit, 11-megapixel CCD camera.

### Preparation of ZnO and TiO_2_ nanoparticle culture media

The ZnO and TiO_2_ nanoparticle culture media were separately prepared with sterile Milli-Q water at a concentration of 1000 mg/L. From each stock solution, aliquots of specific volumes corresponding to the final nZnO or nTiO_2_ concentrations of the working solutions (of 1, 5, 10, 20, 50, 100, 150 and 200 mg/L) were added to each sterile 250 mL flask containing the modified mixed liquor to obtain a final volume of 100 mL and the pH was adjusted to pH 7, using 1.0 M HCl and 1.0 M NaOH (Merck, SA). The use of nanoparticles at concentrations higher than those found in the environment (usually in μg/L)^21^ was due to their lack of effects or impacts on the target isolates during our preliminary analysis (results not reported). To confirm the initial concentrations of nZnO and nTiO_2_ in the modified wastewater mixed liquor medium containing ZnO and TiO_2_ nanoparticles, ICP-OES (AMETEK-Spectro Analytical Instruments GmbH & Co.KG, Germany) was used. The culture medium was autoclaved at 121 °C for 15 min and cooled down to room temperature prior to use. A 1 mL aliquot was plated onto the sterile bacteriological agar and plates were incubated at 30 °C for 24 h to check the sterility of this medium. Only flasks containing the sterile media were inoculated with a known population of the respective test organism isolates. The experimental study was performed in triplicate for each sample to determine the stress effects of nanoparticles on bacterial isolates.

### Experimental study on bioremediation

The experimental study series to determine bioremediation potential were conducted in separate batch reactors, which consisted of 250 mL Erlenmeyer flasks containing 100 mL of the culture media as described by Weber et al.^20^. The culture media were aseptically inoculated with an overnight grown halophilic bacterial isolates (10^2^ cfu/mL). In order to control possible contamination, a culture medium mixed with nanoparticles only and another culture media free of nanoparticles but inoculated with bacterial isolates were used as negative and positive controls, respectively. All the batch reactors were later incubated for five days at a temperature of 30 °C and a shaking speed of 100 rpm. A daily assessment of isolates’ abilities to tolerate the presence of nanoparticles and to remove COD, DO, nitrate and phosphate, were performed. The aliquot samples were filtered using 0.45 μm syringe and the concentrations of nZnO and nTiO_2_ were determined using the ICP-OES (AMETEK-Spectro Analytical Instruments GmbH & Co.KG, Germany). It is worth noting that all of the analyses were carried out in triplicate.

### Statistical analysis

The data were statistically analysed using STATA V13 statistical software. One-tailed T-test was used to was performed to compare the interactions between nZnO and nTiO_2_ and the bacterial isolates in all laboratory batch reactors under various operating conditions (COD, pH, phosphate and nitrate) were also analysed. The interpretation was performed at 95% confidence limit.

### Ethics approval

This article does not contain any studies concerned with the experiment on human or animals.

## Results

### Profile of the wastewater mixed liquor samples

During the study period, the wastewater samples were analysed prior to the preparation and inoculation of the halophilic bacterial isolates. In general, the samples contained a variety of chemical elements such as Ag, Co, Cu, Fe, As, Ba, Li, Ni, Pb and Sr at concentrations of ≤ 2 mg/L, but the concentrations of Na, S, Ca, K and Mg were found to be higher than 15 mg/L.

The Zn and Ti concentrations in the wastewater mixed liquor were ≤ 1 mg/L, the dissolved oxygen in the samples ranged between 0.24 and 0.38 mg/L, while the pH values of the wastewater mixed liquor ranged between 7.33 and 7.77. The average COD concentrations of the samples ranged from 250.6 to 254.5 mg/L. Table 1 summarises the profile of wastewater samples; although the chemical elements appeared to have different concentrations, they did not show a statistically significant difference (*p* > 0.05).

**Table 1:** Profile of the wastewater samples from Daspoort Wastewater Treatment Plant.

	Sample 1	Sample 2	Sample 3	Mean	SD	SA/EPA standard
**Conc. (mg/L)**						
pH	7.33	7.77	7.37	7.49	0.187	5.5–9.5
COD	254.50	250.60	253.70	252.93	1.556	75.00
DO	0.38	0.24	0.28	0.30	0.053	
Ag	1.12	1.13	1.13	1.13	0.004	5
Co	0.37	0.37	0.37	0.37	0.000	0.05
Cu	0.77	0.73	0.73	0.74	0.018	0.01
Fe	0.96	0.94	0.97	0.96	0.011	10
Na	53.91	53.57	56.29	54.59	1.133	100
S	25.17	25.03	25.47	25.22	0.164	200
Ti	1.02	1.02	1.02	1.02	0.000	
Zn	0.41	0.42	0.42	0.42	0.004	0.10
As	0.63	0.64	0.64	0.64	0.004	1
Ba	0.11	0.11	0.11	0.11	0.000	
Ca	65.97	65.46	60.41	63.94	2.358	32
K	15.67	15.56	15.73	15.65	0.062	50
Li	0.10	0.10	0.09	0.10	0.004	
Mg	32.59	33.19	30.53	32.11	1.049	30
Pb	1.93	2.00	1.91	1.95	0.036	0.01
Sr	0.23	0.23	0.23	0.23	0.000	

*SA* South Africa, *EPA* Environmental Protection Agency.

### Characterisation of commercial zinc oxide and titanium dioxide nanoparticles

**Figure 1:**
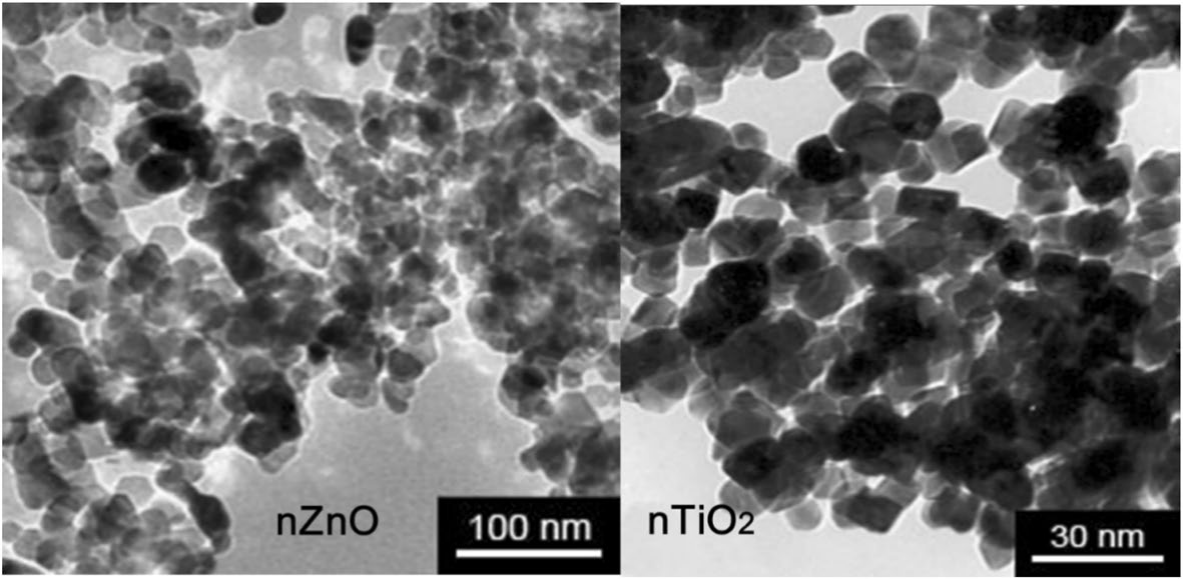
TEM images of zinc oxide and titanium dioxide nanoparticles.

The TEM images of commercial nanoparticles revealed uneven shapes (oval, stretched, circular or even irregular shape) and sizes for both nZnO and nTiO_2_ with the irregular shapes being the main shape for both nanoparticles (Fig. 1). Furthermore, the average crystallite sizes were determined for both nanoparticles, with nTiO_2_ appearing to be wider than nZnO. The particles’ average size distributions were found to range from 25 to 100 nm and 3–30 nm for nZnO and nTiO_2_, respectively. This observation is in line with the manufacturer's specifications, which state that particles should be less than 100 nm in size while, not supplying the size distribution, as well as the prevailing size. The TEM images depicted in Fig. 1, shows that these particles were prone to agglomeration. Further analysis of chosen areas revealed the presence of titanium up to 43% and oxygen up to 31% by weight of dried sample on copper grid, confirming the nanoparticles' composition as nTinO_2_. Similar observation was also observed with zinc up to 50% and oxygen up to 18% confirming the presence of nZnO.

### Effect of nZnO and nTiO_2_ concentrations on pH in culture media inoculated with halophilic bacteria

**Figure 2:**
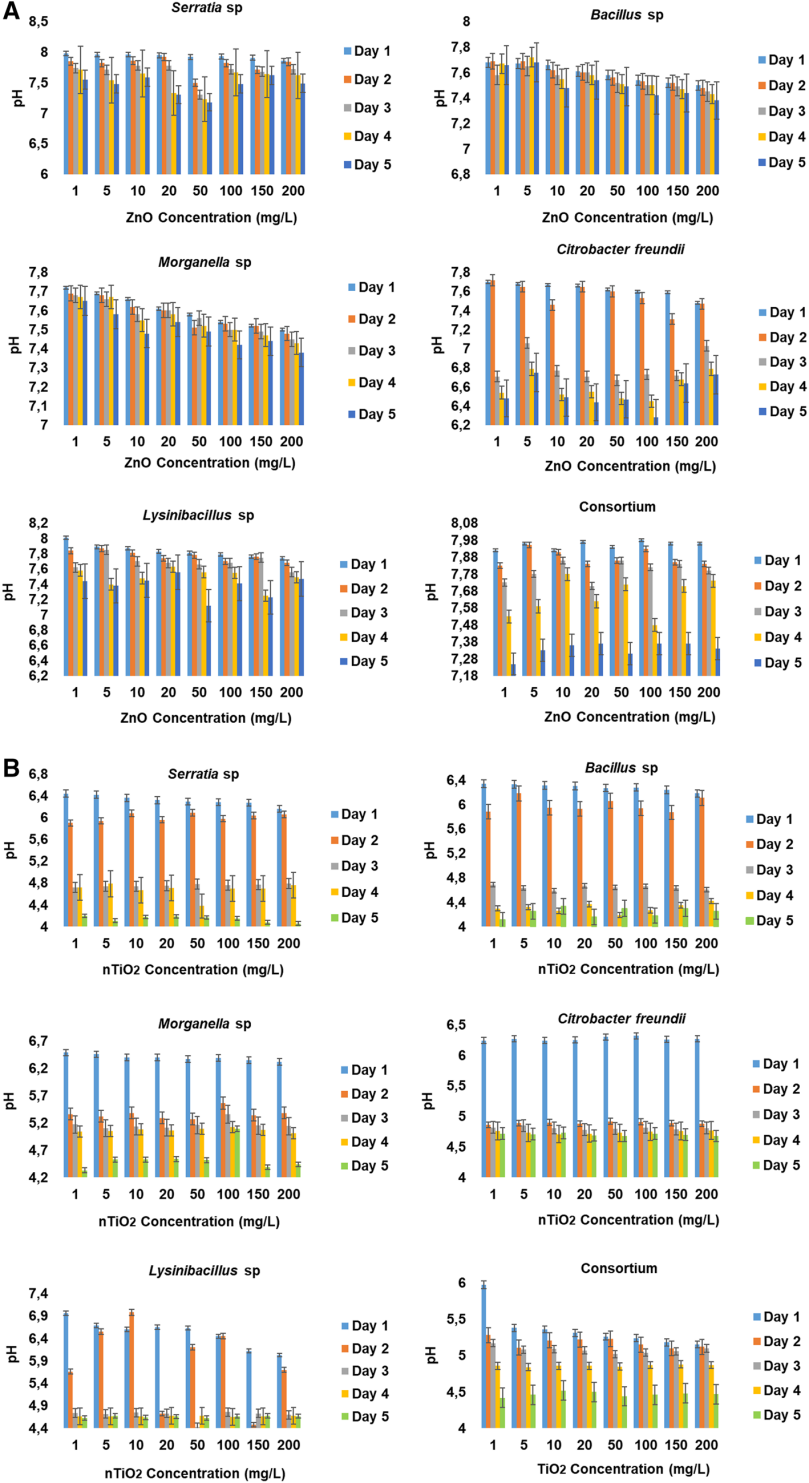
(**A**) Variations in pH during the exposure of halophilic bacteria to wastewater mixed liquor containing various concentrations of zinc oxide nanoparticles. (**B**) Variations in pH during the exposure of halophilic bacteria to wastewater mixed liquor containing various concentrations of titanium dioxide nanoparticles.

Figure 2A,B illustrate the pH variations during the exposure of halophilic bacteria in wastewater mixed liquor media containing various concentrations of zinc oxide nanoparticles. Regardless of the bacterial isolates, in general, the exposure time and nZnO concentrations played a major role in the decrease in pH. Although the critical concentration was at pH 7, an increase in nZnO concentrations from 10 to 50 mg/L triggered a progressive decrease in pH in wastewater mixed liquor inoculated with *Serratia* sp. After a sudden increase in pH that occurred at nZnO concentrations ranging between 100 and 200 mg/L, a progressive decrease in pH was observed throughout the remaining exposure time. For this isolate, the pH of the culture media ranged between 7.86 and 7.98 during the study period.

Culture media inoculated with *Bacillus* sp*.* and *Morganella* sp. exhibited a similar pH pattern characterised by a progressive decrease in pH values with increasing nZnO concentrations. The pH values ranged between 7.38 and 7.68 and between 7.38 and 7.72, respectively. Irrespective of nZnO concentrations in wastewater mixed liquor media inoculated with *Citrobacter freundii*, higher pH values were recorded during the first 2 days of exposure, which progressively decreased during the remaining exposure time. For this isolate, the pH of the culture media ranged between 6.28 and 7.70.

Wastewater mixed liquor culture media inoculated with *Lysinibacillus* sp. were characterised with a pH value of 8.01 in the presence of 1 mg/L nZnO, which progressively decreased as the nZnO concentrations increased throughout the exposure period. Moreover, fluctuations could be observed, which resulted in a pH range of 7.12–8.01. As to the culture media inoculated with the consortium of halophilic bacteria, various nZnO concentrations triggered higher pH values within the first day of exposure. Thereafter, a progressive decrease in pH associated with some fluctuations occurred during the remaining exposure time and the pH values ranged between 7.23 and 7.98.

As shown in Fig. 2B, the presence of nTiO_2_ triggered four different pH activities in wastewater mixed liquor culture media, which were found to be dependent on the particular halophilic bacteria inoculated. It is important to point out that, regardless of the concentrations of nTiO_2_ in the culture media or the inoculated isolates, it was observed that the pH range was still below pH 7. During the exposure period, regardless of the concentrations, nTiO_2_ triggered a similar pH pattern in culture media inoculated with *Serratia* sp. and *Bacillus* sp. This pattern was characterised by pH values of ≤ 6.4 during the first 2 days of exposure, which progressively decreased to ≤ 4.4 during the remaining exposure period. For the culture media inoculated with *Morganella* sp. and *Citrobacter freundii*, irrespective of the concentrations, the nTiO_2_ triggered pH values of ≥ 6.5 during the first day of the exposure period, which progressively decreased to pH 4.3 and 4.6 on the 5th day of exposure, respectively. In the culture medium inoculated with *Lysinibacillus* sp., the concentrations of nTiO_2_ exhibited a pH pattern characterised by fluctuations, although the pH values were found to be higher, particularly during the first day compared to the remaining exposure period. Overall, the pH value ranges were as follows: 4.18–6.34, 4.33–6.49, 4.68–6.24 and 4.63–6.96 for culture media inoculated with *Bacillus* sp., *Morganella* sp., *Citrobacter freundii,* and *Lysinibacillus* sp., respectively. The exposure of a consortium (*Bacillus* sp., *Morganella* sp., *Citrobacter freundii,* and *Lysinibacillus* sp.) of all halophilic bacteria to nTiO_2_ concentrations resulted in the lowest pH values ranging between 5.15 and 5.97 from the first day of the exposure period and progressively decreasing to a range of 4.42–4.50 to the 5th day of exposure.

### Effects of nZnO and nTiO_2_ concentrations on DO uptake efficiency on moderately halophilic bacterial isolates

**Figure 3:**
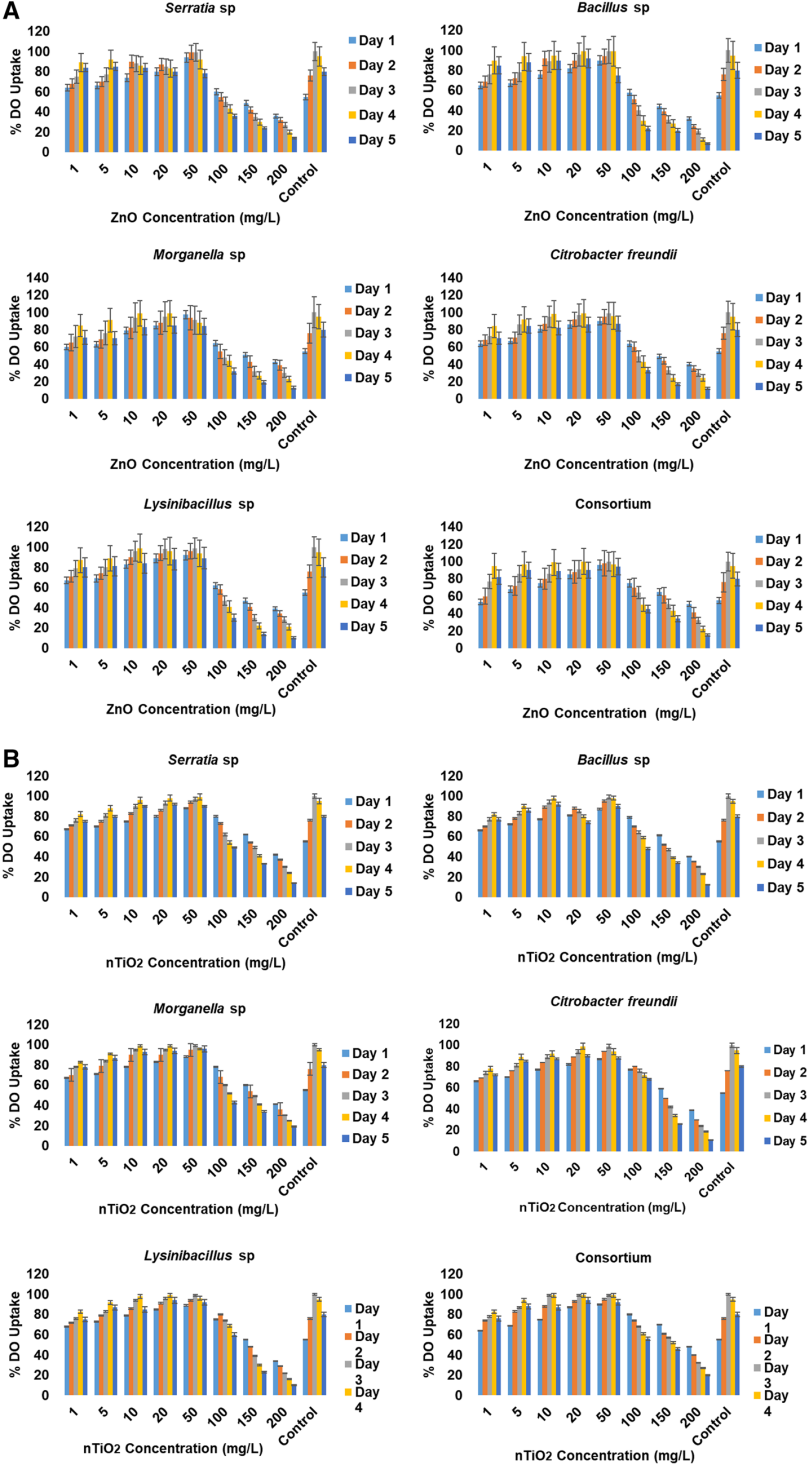
(**A**) Dissolved oxygen uptake (%) by moderately halophilic bacteria under different zinc oxide nanoparticle concentrations in wastewater mixed liquor. (**B**) Dissolved oxygen uptake (%) by moderately halophilic bacteria under different titanium dioxide nanoparticle concentrations in wastewater mixed liquor.

Figure 3A,B depict the uptake of DO by moderately halophilic bacterial isolates exposed to wastewaters containing different nZnO and nTiO_2_ concentrations as well as to wastewaters without the target metal oxide nanoparticles. In general, all target halophilic bacteria were able to gradually uptake nZnO and nTiO_2_ in concentrations varying from 1 to 50 mg/L with higher DO uptake recorded in the presence of the former rather than the latter concentration. Low DO uptake was observed on day 1 and day 2 of the isolates’ exposure, regardless of the type of metal oxide nanoparticles. A significant DO uptake was observed specially in the presence of 50 mg/L on days 3 and 4 (DO uptake of up to 100% for nZnO and 99% for nTiO_2_). The target metal oxide nanoparticles at concentrations ranging between 100 and 200 mg/L triggered a significant gradual decrease in DO uptake (up to 10% under both metal oxide nanoparticles) in wastewater mixed liquor media, while the DO uptake in the control (which contained bacteria within a nanoparticle-free medium) ranged from to 60 to 80% with the highest observed on the 4th day at 100%.

In the presence of nZnO, the highest DO uptake (100%) was observed with the bacterial consortium on day 3 at a concentration of 50 mg/L and on day 4 at 20 mg/L, while *Lysinibacillus* sp. exhibited the lowest DO uptake at 10%. In the presence of nTiO_2_, the DO response was lower compared to that of nZnO, with the highest uptake observed with the consortium (100%) from the 3rd and 4th days of the experiment at a concentration range of 10–50 mg/L, followed by *Morganella* sp. (99%), and the lowest response was demonstrated by *Lysinibacillus* sp. (10%). The control, which consisted of the bacterial consortium in the presence of a nanoparticle-free medium, was shown to have a better response with the highest DO uptake (100%) observed on the 3rd day compared to the medium containing nTiO_2_.

### Effects of nanoparticle concentrations on COD removal efficiency of moderately halophilic bacterial isolates

In general, halophilic bacterial isolates exhibited a low COD removal efficiency in the presence of low concentrations (1–10 mg/L) of both metal oxide nanoparticles compared to the control (free of nanoparticles, inoculated with the target isolates), which had a rather better response for COD removal, up to 85%. Although COD removal was observed at lower concentrations, COD release was also observed at higher concentrations in the presence of both metal oxide nanoparticles. In the presence of nTiO_2_, a higher release of up to − 60% was observed, specifically with *Citrobacter freundii*, which showed a complete COD release in nTiO_2_ concentrations ranging from 1 to 200 mg/L, compared to exposure to varying nZnO concentrations where a COD release of up to − 21% was observed.

**Figure 4:**
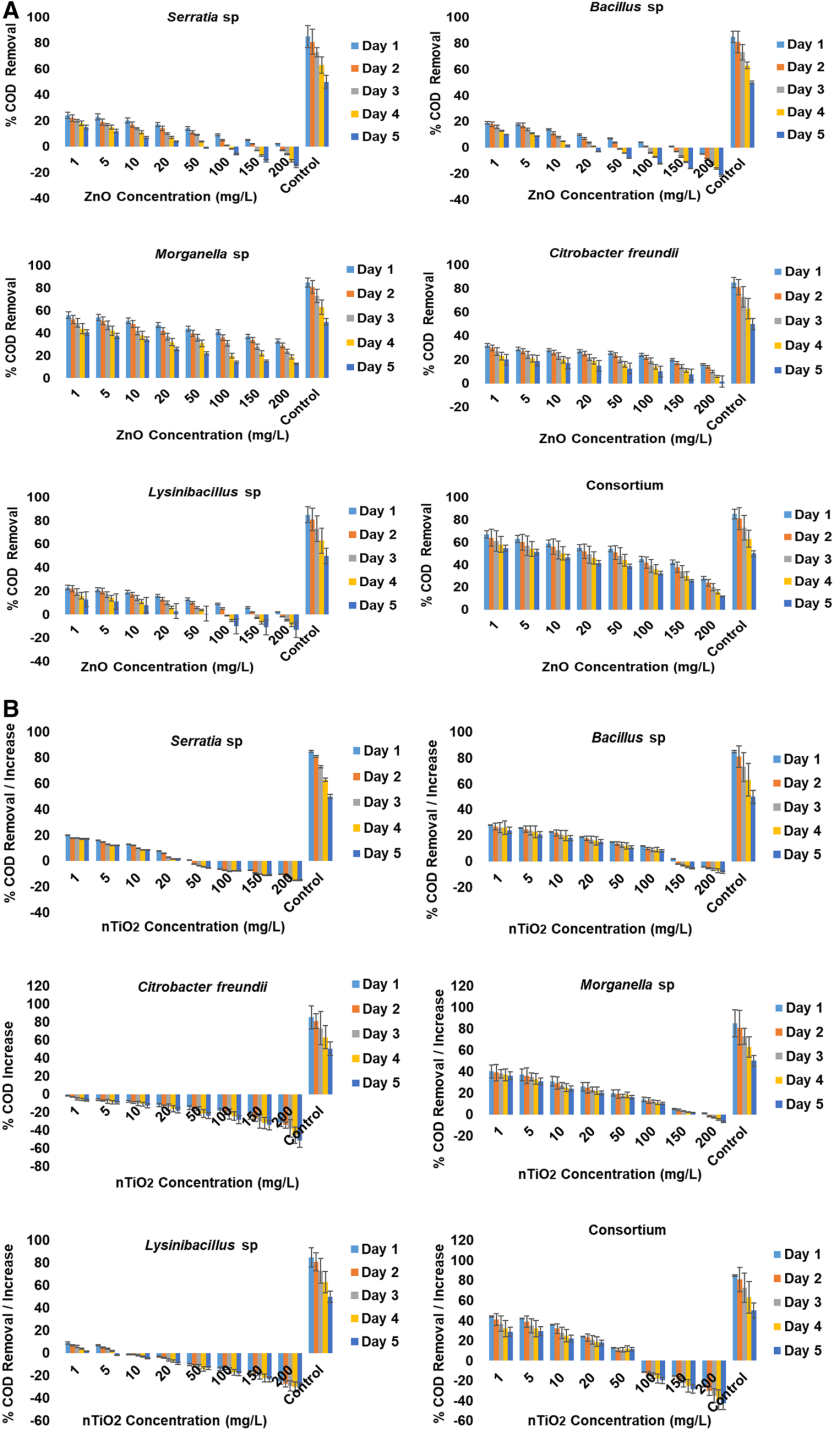
(**A**) Representation of COD removal/increase (%) in different nZnO concentrations in modified wastewater mixed liquor. (**B**) Representation of COD removal/increase (%) in different nTiO_2_ concentrations in modified wastewater mixed liquor.

Three different patterns were identified in terms of the effect of nZnO on COD removal efficiency, and are summarised as follows: (1) *Serratia, Bacillus* and *Lysinibacillus* sp. were able to remove approximately 20% COD on the first day and the second day of their exposure to 1 mg/L and 5 mg/L nZnO. Thereafter, a gradual decrease in COD removal was associated with the extended exposure time and also with an increase in nZnO concentrations, which resulted in the release of COD in the culture medium of up to ≥ − 21%. This release was species-specific and apparent in the presence of 100–200 mg/L nZnO for *Serratia* and *Lysinibacillus* spp. and 50–200 mg/L nZnO for *Bacillus*. (2) Irrespective of nZnO concentrations, *Morganella* sp. exhibited the capability to remove up to 56% COD from the culture medium containing initial concentrations of 1–10 mg/L nZnO during the first day of exposure. Its capability for COD removal gradually decreased when exposed to a concentration range of 50–200 mg/L nZnO during the extended exposure period. A similar pattern was also observed with *Citrobacter freundii* on the first day with a removal capability of ≤ 40 mg/L COD, which gradually decreased over extended exposure time with an increase in nZnO concentration. (3) Finally, the consortium of the target moderately halophilic bacteria, increased their COD removal efficiency up to 67% when they were exposed to culture media with 1 mg/L nZnO; thereafter, a gradual decrease occurred with extended exposure time and also with increasing nZnO concentrations. Statistically, significant differences in COD removal were observed between the consortium and the individual bacteria (*p* < 0.05), except with *Morganella* sp. A significant difference in COD removal was also noted between this bacterial species and the other three halophilic bacteria (Fig. 4A).

In the presence of nTiO_2_, the COD removal efficiency of the selected halophilic bacteria was found to be more species-specific and lower compared to nZnO. *Serratia* sp. was capable of removing 20% of the COD from the culture medium containing 1 mg/L nTiO_2_. Gradual decreases in COD removal efficiency occurred with extended exposure periods and an increase in nTiO_2_ concentration up to 20 mg/L. At a concentration ranging between 50 and 200 mg/L nTiO_2_, a progressive release of the COD was observed in the culture medium. A similar pattern was also observed with *Bacillus* sp., which was able to remove up to 35% of COD from the culture medium containing a concentration range of 1–5 mg/L nTiO_2_ during the first day. Thereafter, a progressive decrease in its efficiency was noted at concentrations of between 10 and 100 mg/L nTiO_2_ until the COD release occurred in the medium with the highest nTiO_2_ concentrations of 150 and 200 mg/L. All the various concentrations of nTiO_2_ had a negative effect on *Citrobacter freundii* with no COD removal noted during the exposure time. *Morganella* sp. was found to be the only individual isolate capable of removing up to 40% of COD from the medium containing the initial concentration range of 1–5 mg/L nTiO_2_ within 1 day of exposure. Subsequently, a gradual decrease of its COD removal efficiency was apparent with an increasing nTiO_2_ concentration in the culture media containing 10–150 mg/L nTiO_2_, while the release of COD (− 7%) occurred back in the culture medium with 200 mg/L with an extended exposure time. Less than 10% of COD was removed by *Lysinibacillus* from wastewater mixed liquor containing a concentration range of 1–5 mg/L nTiO_2_. With the extended exposure time and increasing nTiO_2_ concentrations (10–200 mg/L) in the culture medium, the release of COD took place up to > − 20%. The consortium of halophilic bacteria exhibited COD removal up to ≤ 42% from wastewater mixed liquor containing 1–5 mg/L nTiO_2_ within the first day of the exposure time. While a gradual decrease in its efficiency was apparent in the culture media with 10–50 mg/L nTiO_2_, the nTiO_2_ concentrations of 100–200 mg/L triggered the release of COD in the medium (Fig. 4B).

### Effects of nanoparticle concentrations on nutrient removal efficiency of moderately halophilic bacterial isolates

The halophilic bacterial isolates exhibited different phosphate removal efficiency patterns in the presence of both metal oxide nanoparticles, which ranged from 25 to over 65% in the presence of nZnO and from 30 to over 70% in the presence of nTiO_2_. The removal efficiency decreased as the concentration of the metal oxide nanoparticles increased. In the control (free of nanoparticles, but inoculated with the target isolates), a removal response of up to 72% was recorded.

Four different phosphate removal patterns could be identified in the presence of various concentrations of nZnO, and are summarised as follows: (1) *Serratia* and *Bacillus* sp*.* were able to remove approximately < 40% of phosphate when exposed to the concentration range of 1–5 mg/L nZnO and < 50% of phosphate when exposed to 10–20 mg/L on the first and second day, respectively. Thereafter, a gradual decrease in phosphate removal was associated with the extended exposure time after the 3rd day. (2) *Morganella* sp. was able to remove slightly more than 40% of phosphate on the first and second day when exposed to the concentration range of 1–20 mg/L nZnO; on the first and second day, a removal of approximately 40% could still be observed at nZnO concentrations from 20 to up to 100 mg/L. Thereafter, from 150 to 200 mg/L, a gradual decrease was observed over the extended exposure time. (3) *Citrobacter freundii* were able to remove approximately 25% of phosphate on the first day and approximately 20% on the second day of their exposure to 1 mg/L and 1–5 mg/L nZnO, respectively. Thereafter, a gradual decrease in phosphate removal was associated with the extended exposure time with the lowest decrease up to < 1% apparent when exposed to 200 mg/L nZnO. (4) Finally irrespective of nZnO concentrations, *Lysinibacillus* sp. and the bacterial consortium exhibited the capability to remove approximately > 65% of phosphate from the culture medium containing initial concentrations of up to 1–5 mg/L nZnO during the first day of exposure. Its capability for phosphate removal gradually decreased when exposed to higher nZnO concentrations during the extended exposure period.

**Figure 5:**
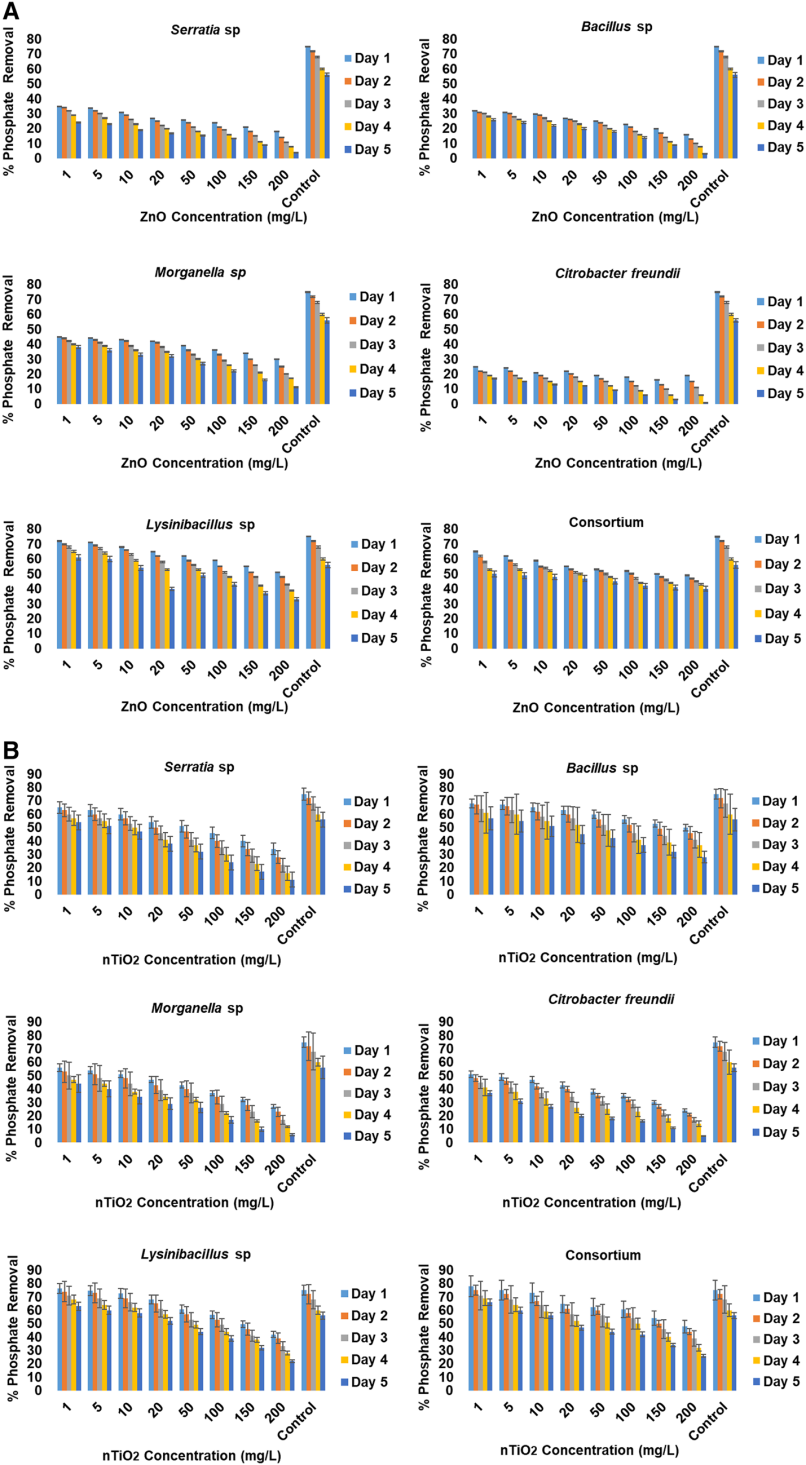
(**A**) Representation of percentage removal of phosphate in different nZnO concentrations in modified wastewater mixed liquor. (**B**) Representation of percentage removal of phosphate in different nTiO_2_ concentrations in modified wastewater mixed liquor.

Statistically, significant differences in phosphate removal were noted between the consortium and individual bacteria (*p* < 0.05), except for *Lysinibacillus* sp. A significant difference in phosphate removal was also noted between this bacterial species and the other three halophilic bacteria (Fig. 5A).

In the presence of various concentrations of nTiO_2_, the phosphate removal efficiency of the selected halophilic bacteria was found to be higher compared to nZnO, and four different patterns could be identified, summarised as follows: (1) *Serratia* and *Bacillus* sp were able to remove a low concentration of phosphate at the initial stages of exposure. Aproximately < 40% of phosphate was removed when exposed to the concentration range of 1–5 mg/L nZnO in the presence of *Serratia* sp. And < 50% of phosphate when exposed to 10–20 mg/L in the presence of *Bacillus* sp on the first and second day, respectively. Thereafter, a gradual decrease in phosphate removal was associated with the extended exposure time after the 3rd day. A decrease in phosphate removal was observed upon exposure to a concentration range of 50–200 mg/L for *Serratia* sp. after the 3rd day. However, for *Bacillus* sp*.*, a gradual decrease in phosphate removal throughout was associated with the extended exposure time. (2) *Morganella* sp. was able to remove slightly more than 50% of phosphate on the first and second day when exposed to the concentration range of 1–10 mg/L nTiO_2_; on the first and second day phosphate removal of approximately 40% could still be observed upon exposure to a concentration range of 20–50 mg/L nTiO_2_ on the 1st and 2nd day. Thereafter, from 50 to 200 mg/L, a gradual decrease was observed over the extended exposure time. (3) *Citrobacter freundii* were able to remove over 45% of phosphate on the first and second day and approximately 30% on the first and second day of their exposure to concentrations ranging from 1 to 10 mg/L and 10–20 mg/L nZnO, respectively. Thereafter, a gradual decrease in phosphate removal was associated with the extended nTiO_2_ exposure time (50–200 mg/L). (4) Lastly; irrespective of nTiO_2_ concentrations, *Lysinibacillus* sp. and the bacterial consortium exhibited the capability to remove approximately over 70% of phosphate from the culture medium containing initial concentrations of up to 1–50 mg/L nZnO during the first and second day of exposure. Thereafter, a slight gradual decrease was observed when the isolates were exposed to higher nTiO_2_ concentrations during the extended exposure period. The control (containing bacteria in nTiO_2_-free concentrations) appeared to have a higher phosphate removal efficiency of approximately 80% (Fig. 5B).

**Figure 6:**
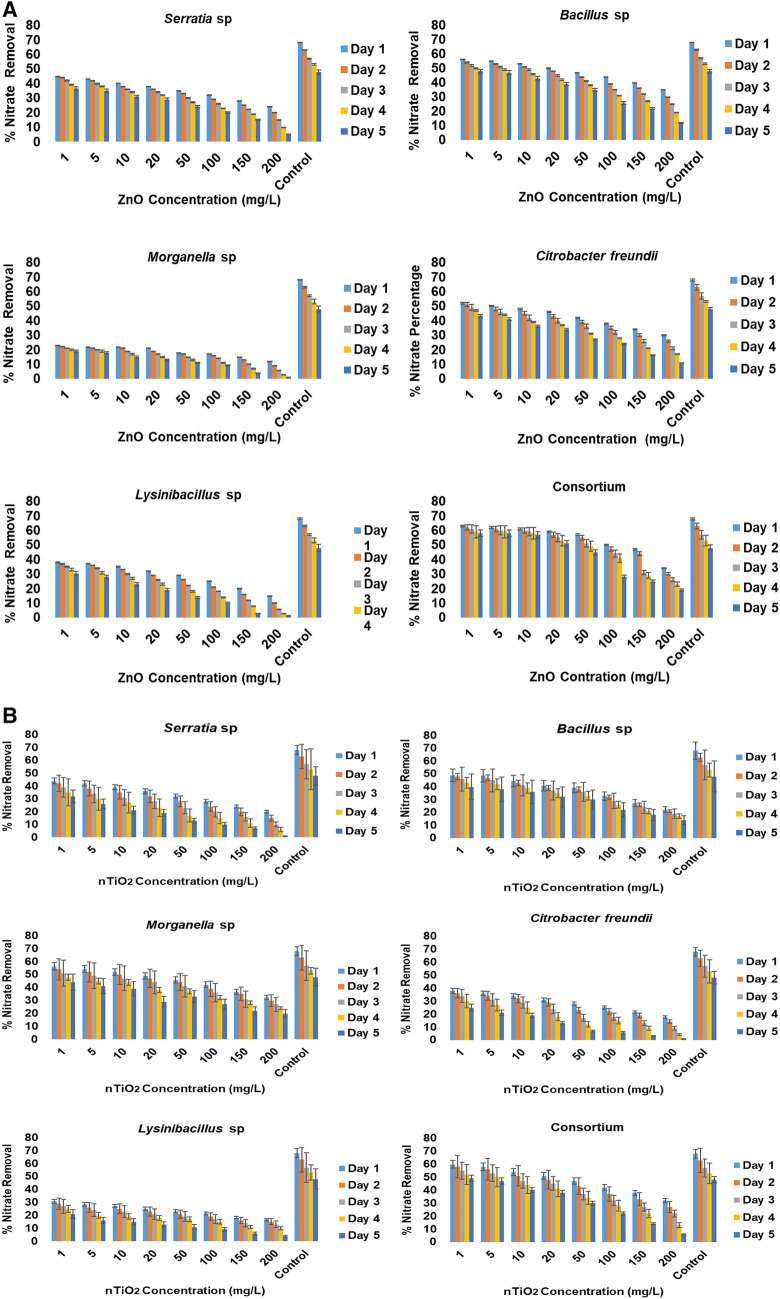
(**A**) Representation of percentage removal of nitrate in different nZnO concentrations in modified wastewater mixed liquor. (**B**) Representation of percentage removal of nitrate in different nTiO_2_ concentrations in modified wastewater mixed liquor.

In terms of nitrate removal, the halophilic bacterial isolates showed an individual nitrate removal pattern, generally similar to phosphate removal, when exposed to both metal oxide nanoparticles. A maximum nitrate removal of approximately 70% was observed in the control (free of nanoparticles, but inoculated with the target isolates). The nitrate removal efficiency of halophilic bacterial isolates upon exposure to nZnO was observed to have single patterns as the isolates each showed an individual removal pattern: (1) *Morganella* sp. was found to be the isolate with the lowest nitrate removal efficiency, with approximately 20% removal when exposed to 1–20 mg/L nZnO on the first and second day of exposure. A nitrate removal below 20% was recorded when this bacterium was exposed to 50–20 mg/L nZnO, followed by a progressive decrease as nZnO concentrations increased. (2) *Lysinibacillus* sp. exhibited the second lowest nitrate removal efficiency, with over 35% removal when exposed to 1–10 mg/L nZnO on the first and second days. This was followed by a progressive decrease as the nZnO concentration increased from 20 to 200 mg/L over time. (3) *Serratia* sp. achieved a nitrate removal of over 40% when exposed to 1–5 mg/L nZnO on the first and second days. A removal of below 40% was detected in the concentration range of 10–50 mg/L nZnO, which was followed by a gradual decrease in removal efficiency as nZnO concentrations increased over time. (4) *Citrobacter freundii* was observed to have a higher nitrate removal efficiency of over 50% upon exposure to the concentration range of 1–10 mg/L nZnO on the first and second day, compared with the preceding isolates on the first and second day. Thereafter, a removal of ≤ 40% in the concentration range of 20–50 mg/L nZnO was observed, which was followed by a decrease as the nZnO concentrations increased over the exposure time. (5) *Bacillus* sp. showed a removal of approximately 55% when exposed to a concentration range of 1–10 mg/L nZnO on the first to the third day, followed by a removal of approximately 50%, which was followed by a progressive decrease as nZnO concentrations increased over the exposure time. (6) The consortium of isolates represented the highest nitrate removal with approximately over 60% removal efficiency upon exposure to the concentration range of 1–20 mg/L nZnO from the 1st to the 4th day, and a removal below 60% as the concentration increased over the time of exposure (50–200 mg/L). Statistically, significant differences in nitrate removal were noted between the consortium and individual bacteria (*p* < 0.05) (Fig. 6A).

In the presence of nTiO_2_ (Fig. 6B), the bacterial isolates showed a similar nitrate removal pattern and efficiency in comparison to nZnO. Each isolate showed the following nitrate removal efficiency: (1) *Lysinibacillus* sp. represented the isolates with the lowest nitrate removal efficiency with approximately 30% removal in concentrations of between 1 and 10 mg/L nTiO_2_ on the first and second days of exposure and a removal of approximately 35% in the concentration range of 20–100 mg/L nTiO_2_, followed by a decrease as nTiO_2_ concentrations increased (150–200 mg/L) over time. (2) *Citrobacter freundii* achieved a nitrate removal efficiency of approximately 40% in the concentration range of 1–5 mg/L nTiO_2_ on the first and second days of exposure and of ≤ 40% compared with the preceding isolates on the first and second days. Thereafter, a removal ≤ 40% in the concentration range of 10–20 mg/L nTiO_2_ was observed, which was followed by a decrease as the nTiO_2_ concentrations increased (50–200 mg/L) over the exposure time. (3) *Serratia* sp. achieved a nitrate removal of over 40% in the TiO_2_ concentration range of 1–5 mg/L on the first and second days of exposure and a removal of approximately 40% was observed in the concentration range of 10–20 mg/L, followed by a gradual removal efficiency decrease, as nTiO_2_ concentrations increased (50–200 mg/L) over time. iv) *Bacillus* sp., showed a nitrate removal of approximately 50% in the presence of 1–10 mg/L nTiO_2_ on the first to the second days, followed by a removal of approximately 45%, which was followed by a progressive decrease as nTiO_2_ concentrations increased (100–200 mg/L) over the exposure time. (5) *Morganella* sp. showed a nitrate removal of approximately 55% upon exposure to 1–5 mg/L nTiO_2_ on the first to the second days, followed by a removal of approximately 50% (10–20 mg/L), which progressively decreased as nTiO_2_ concentrations increased (50–200 mg/L) over the exposure time. (6) Lastly, the consortium of isolates exhibited the highest nitrate removal in the presence of nTiO_2_, with approximately < 60% removal efficiency upon exposure to the concentration range of 1–5 mg/L nTiO_2_ from the first day to the second day, and a removal of below 60% at the concentration of 10–20 mg/L nTiO_2_. Thereafter, a gradual decrease in its nitrate removal efficiency was apparent with increasing TiO_2_ concentrations in the culture media containing 50–200 mg/L nTiO_2_. Statistically, significant differences in nitrate removal were noted between the consortium and individual bacteria (*p* < 0.05) (Fig. 6B).

## Discussion

The rapid advances in nanotechnology and the growing number of applications of metal oxide nanoparticles as well the concomitant increase in the amount of nanomaterials being discharged into the environment constitute a great risk for the environment as some of them are not easily biodegraded. The report by Włodarczyk and Kwarciak-Kozłowska^22^ indicated that nanoparticles are manufactured annually in the cosmetic and medical products industry and were estimated to amount to about 58 thousand tons between 2011 and 2020. Even though nanoparticles such as zinc oxide and titanium oxide have properties that are beneficial for product improvement in most fields, little is done on how to handle these products and this could have a detrimental effect on the environment once discharged^23–25^. South Africa, like the rest of the world, has been increasingly using the benefits of the use of nanoparticles in most domains such as biomedical applications, medicine, paints, wastewater treatment, etc. Numerous research reports have revealed that, worldwide, the increase in the use of engineered nanoparticles can lead to their release into the environment and thereafter it may become a public health concern^26–28^. Therefore, assessing and managing the possible risks associated with the production and usage of nanomaterials will reduce the risks of exposure to toxic compounds in the environment, animals and humans. Hence, this study investigated the ability of moderately halophilic bacterial isolates (*Serratia* sp., *Bacillus* sp., *Morganella* sp., *Citrobacter freundii* and *Lysinibacillus* sp.) to treat polluted wastewater in the presence of nZnO and nTiO_2_ nanoparticles. For this purpose, the first phase of the study consisted to determine the physicochemical profile of wastewater samples. The results revealed the presence of metals, metalloids and non-metals at concentrations beyond the permissible limit for South African wastewater effluents: 75 mg/L, 0.05 mg/L, 0.01 mg/L, 0.1 mg/L 0.01 mg/L for COD, Co, Cu, Zn and Pb, respectively. In spite of their potential toxicity, the permissible limits for Fe, Na, S, Ti, As, Ba, Ca, K and Sr in effluents of small wastewater treatment works are still not available in the design guidelines issued by the Department of Public Works, South Africa^29^ and national regulatory standards issued by the USEPA^30^. The pH and Ni of the wastewater samples were found to be within the recommended range of 5.5–9.5 and 0.2 mg/L, respectively.

The first stage was followed by the characterisation of both nZnO and nTiO_2_ in terms of their shapes and sizes using TEM, which demonstrated that these commercial nanoparticles were polydisperse with regular and irregular shapes and with average crystallite sizes (Fig. 1). Previous investigators have also observed the inhomogeneity in nZnO^31,32^ and nTiO_2_^33,34^ nanoparticle size and the crystal shape. According to Yang et al.^31^, the crystal shape of metal oxide nanoparticles such as ZnO NPs plays a major role irrespective of the size of the particles.

The results of the present study showed that the growth of the target halophilic bacterial isolates in the presence of various concentrations of nZnO and nTiO_2_ was concentration-dependent, irrespective of pH. Results clearly revealed that both metal oxide nanoparticles also impacted the pH of wastewater. In spite of the pH variations that occurred in wastewater media inoculated with individual halophilic bacteria, the presence of both nZnO and nTiO_2_ had a significant impact on pH values. At a low nZnO concentration, the wastewater pH increased from the initial pH value of 7 up to 8.1 and a progressive decrease in pH values was noted with an increasing concentration of nZnO. As can be seen from Fig. 2A, this means that there was no definitive pH range for various biological activities of the target halophilic bacteria as the pH fluctuated between 6 and 8.1^35^, especially in wastewater (pH 7.18–7.98) inoculated with a consortium of halophilic bacteria. Instead of any pH increase in wastewater inoculated with the individual halophilic bacterial isolates, the nTiO_2_ triggered a significant decrease in pH from 6.9 down to 4 with a pH range of 4–7 recorded due to increasing nTiO_2_ concentrations (Fig. 2B). In wastewater inoculated with a consortium of halophilic bacteria a pH range of 6–4 was observed, which was also due to an increasing concentration of nTiO_2_ during the exposure time. This trend clearly showed the lack of a definitive pH range for various biological activities as the presence of nTiO_2_ presented a lower range^35^. It was also argued that the decrease of pH in the media could have also be caused by fermentation due to the concomitant decrease of DO. Though these authors used another nanoparticle, it was noted that the decrease of pH favoured the adsorption of silver nanoparticle on the bacterial cell surface^36^.

In addition to pH, the DO is among the factors influencing the growth of microorganisms in wastewater systems. For both metal oxide nanoparticles, a significant DO uptake was observed in wastewaters containing 1–50 mg/L, a concentration range which triggered the DO uptake of up to 100% for nZnO and 99% for nTiO_2_. However, their significant toxicity effects could be noted when all test isolates were exposed to the target metal oxide nanoparticles at concentrations ranging between 100 and 200 mg/L, which triggered a significant gradual decrease in DO uptake compared to the lower concentrations (Fig. 3A/B). The toxicity effect of nZnO and nTiO_2_ on the target moderately halophilic bacteria occurred at higher concentrations (100 mg/L and 200 mg/L), which negatively impacted on halophilic bacterial growth and consequently on the decrease of their ability for DO uptake. The detrimental effects of commercial zinc oxide on bacterial growth and DO uptake have been previously demonstrated by Mboyi et al.^32^ in their study on *Bacillus licheniformis, Brevibacillus laterosporus* and *Pseudomonas putida*. These authors also pointed out that the uptake of DO was strongly linked to bacterial growth as no uptake was observed in the absence of bacterial growth. Furthermore, they found that DO uptake and bacterial growth decreased significantly (*p* < 0.05) with increasing concentrations of nanomaterials in the media. The decreasing rate of oxygen removal could be caused by the saturation of the bacterial isolates in taking up the nanoparticles and the time of exposure (5 days) being too short for the re-adaptation and recovery of the bacterial isolates. According to Spietz et al.^37^, the decrease of DO itself from the environment was responsible for the negative impact of bacterial richness and growth. The decrease of bacterial growth was found not to be linked to the salt content of the media since the growth in the free-NP media did not show a significant decrease (Fig. S1). Results of this study are in agreement with those reported by a number of authors^15,38^ who reported that the oxygen uptake of *Pseudomonas putida* was stimulated when inoculated in diluted industrial effluent but was inhibited in highly polluted industrial wastewater^39^.

The present study further investigated the ability of bacterial isolates in removing COD, phosphate and nitrate from wastewater mixed liquor culture media, as their presence has the ability to impact on the growth of microorganisms. The COD test is commonly used to indirectly measure the amount of organic compounds in water. It is a measure of the oxygen equivalent of that portion of the organic matter in a sample that is susceptible to be oxidised by a strong chemical oxidant. The COD removal efficiency was very low in the presence of both metal oxide nanoparticles. A significant uptake was observed to be species-specific in the presence of both metal oxides, while an increase in COD was observed in both media containing nZnO and nTiO_2_. However, the significant toxicity effects triggered by their increased concentrations could be noted at a wider range for the test isolates in the wastewater containing the nTiO_2_ concentration range (1–200 mg/L) compared to the nZnO concentration range (50–200 mg/L) (Fig. 4A,B). These results could be explained by the fact that the carbon source used in this experimental study was sucrose and this was found to be more favourable to the halophilic bacteria compared to glucose, which is the common carbon source used in complex hypersaline media^40,41^. In other words, the composition of the mixture of COD medium, mixed liquor and the sucrose (2%), created a toxic solution. Although sucrose was found to be the preferred carbon source of the moderately halophilic bacteria at their optimum growth conditions, it affected the composition of the industrial mixed liquor, and its high concentration (2%) in the mixed liquor in the presence of nZnO and nTiO_2_ had a negative impact on halophilic bacterial growth. Consequently, on their ability to remove COD, which turned out to be lower compared to other findings, such as those of Dan et al.^42^ who showed that halophilic bacteria are able to remove COD at high percentages, and those of Uygur and Kargi^43^ who evaluated a culture of *Zoogloea ramigera* and *Halobacterium halobium* and reported COD removal efficiency of about 77% at 1% salt in a mixed culture in an activated sludge process.

Dissolved oxygen was also linked to COD removal, as no uptake was observed in the absence of bacterial growth. As mentioned by Uygur and Kargi^43^ in their study, in order to obtain equivalent COD removal efficiency, lower F/M ratios were required at higher salt contents for both yeast and bacterial sludge. This finding validates the fact that bacterial growth decreased significantly (*p* < 0.05) with increasing nanoparticle concentrations in the media. Woolard and Irvine^16^ also demonstrated that halophilic bacteria have the ability to remove COD; *Halobacterium* obtained the highest efficiency at 1% salt concentration and COD removal of 70–80% at salt concentrations of 4–5%.

In wastewater containing nZnO or nTiO_2_ concentrations of between 1 and 50 mg/L, the test isolates demonstrated a significant phosphate and nitrate removal efficiency, as follows: at this concentration range, phosphate removal of up to 70% for media containing nZnO and 78% for media containing nTiO_2_, and nitrate removal of up to 65% for media containing nZnO and 62% for media containing nTiO_2_. Nevertheless, all the test isolates exhibited significant toxicity effects, which were noted when exposed to high concentrations (100–200 mg/L) of the target metal oxide nanoparticles, and consequently triggered a significant progressive decrease in phosphate removal compared to the lower nanoparticle concentrations (Figs. 5A/B, 6A/B). These results validate a significant decrease in halophilic bacterial growth, as the toxicity effect of nZnO and nTiO_2_ on the target moderately halophilic bacteria occurred at higher concentrations (100 mg/L and 200 mg/L) and also as a result of the composition of the wastewater media (sucrose at 2% being the preferred carbon source of the moderately halophilic isolates), therefore, decreasing the ability of microorganisms to break down excessive nitrate and phosphate, and also negatively impacting their ability to remove phosphate. These findings are validated by studies conducted by Ekama et al.^44^ and Rybicki^45^, who have illustrated that phosphate removal is impacted by the availability of easily biodegradable carbon sources, needed by phosphorus-storing microorganisms in relation to the amount of phosphorus that must be removed. In testing the influence of carbon source on nitrate removal of polluted groundwater in a denitrifying submerged filter, Gomez et al.^46^ reported that sucrose was the least efficient carbon source in comparison with ethanol and methanol as carbon sources.

## Conclusion

The present study evaluated the capability of moderately halophilic bacterial isolates (*Serratia* sp.,* Bacillus* sp.,* Morganella* sp.,* Citrobacter freundii* and *Lysinibacillus* sp.) to treat a polluted wastewater in the presence of zinc oxide and titanium dioxide nanoparticles. In the presence of test nanoparticles, the growth of moderately halophilic bacteria was found to be significantly affected by the increasing nanoparticle concentrations ranging between 50 and 200 mg/L. In contrast, lower concentrations (1–20 mg/L) of both nZnO and nTiO_2_ resulted in a significant increase in bacterial growth. The changing pH values of the media did not significantly affect the growth of bacterial isolates. However, the changing concentration of nZnO and nTiO_2_ impacted on the wastewater pH with higher concentrations triggering a progressive decrease in pH values. Similar observations were observed for DO uptake, COD and nutrient removal. It can be concluded that moderately halophilic bacteria such as *Serratia* sp., *Bacillus* sp., *Morganella* sp., *Citrobacter freundii*, *Lysinibacillus* sp. and a consortium of these organisms can be employed for the bioremediation of wastewaters contaminated with low nZnO and nTiO_2_ concentrations.

## Supplementary Information

Supplementary Figures.
