# Supplementary material for: Comparing the effect of zinc oxide and titanium dioxide nanoparticles on the ability of moderately halophilic bacteria to treat wastewater
Source: Sci Rep. 2021 Aug 20;11:16969. doi: 10.1038/s41598-021-96413-5 (PMC8379202; doi:10.1038/s41598-021-96413-5)
Supplement: Supplementary file 1 — Supplementary Figures. [file 41598_2021_96413_MOESM1_ESM.docx]

Supplementary materials


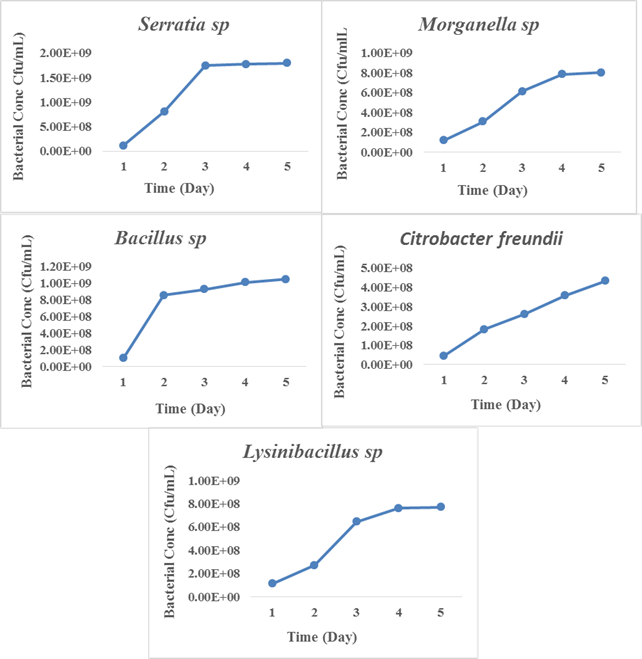


Figure S1: Average growth curve of moderately halophilic bacteria isolates in free nanoparticles medium. Containing wastewater mixed liquor medium at the isolates optimum condition (30 °C, 4 % NaCl, 2 % Sucrose)


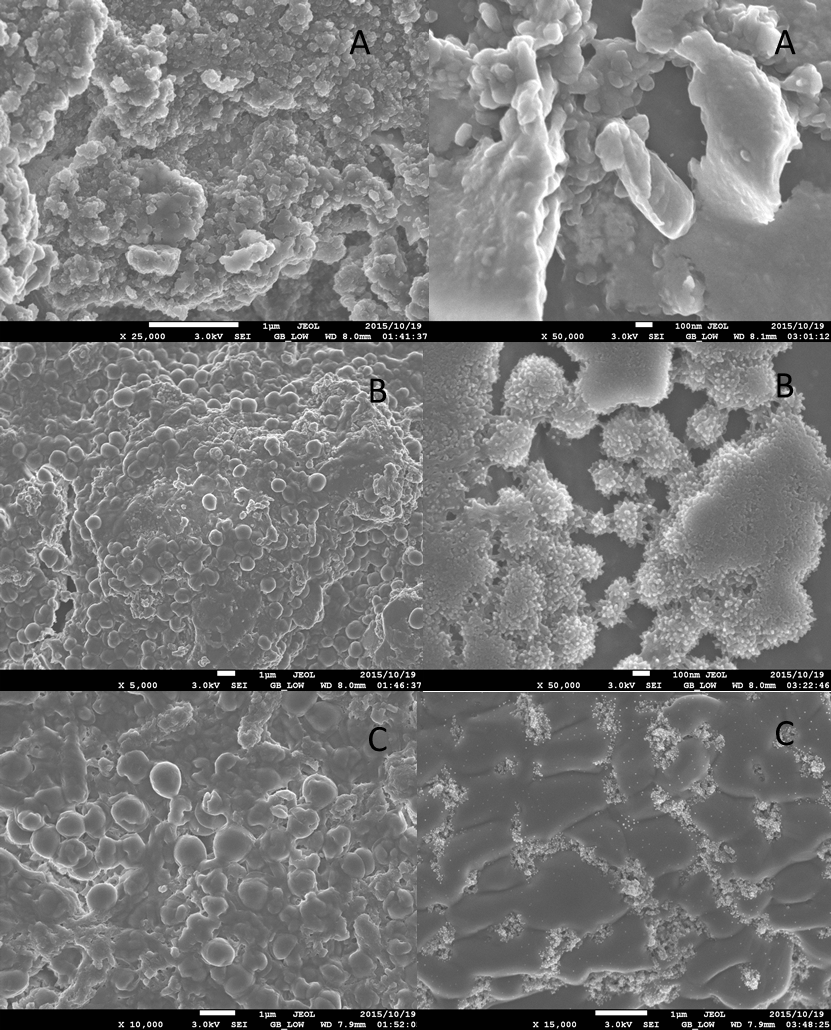


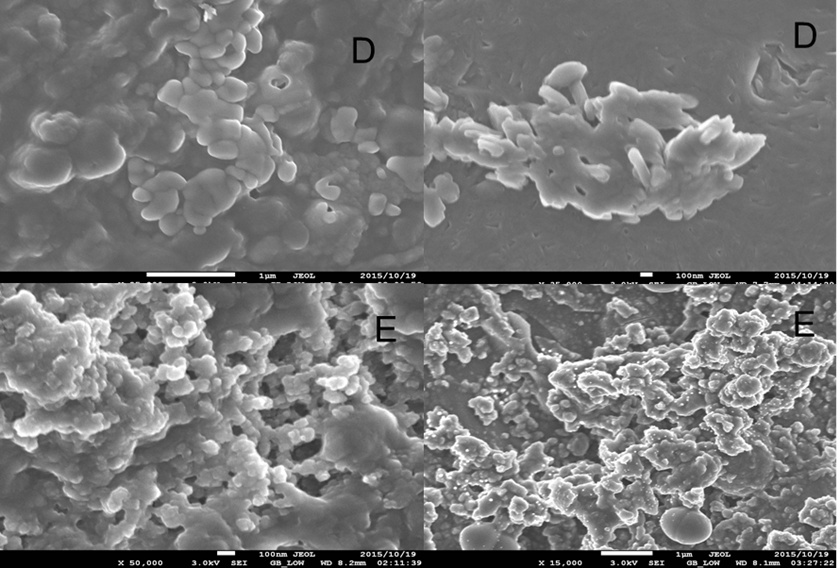


**Figure S2**: **Representation of SEM analysis, on the left halophilic bacteria isolates in free presence of Zinc oxide, on the right halophilic bacteria in presence of 50ppm ZnO. A: *Serratia sp* (1µm) in free ZnO- (100nm) in presence of ZnO. B: *Bacillus sp* (1µm) in free ZnO- (100nm) in presence of ZnO. C: *Morganella sp* (1µm) in free ZnO- (1µm) in presence of ZnO. D: *Citrobacter freundii* (1µm) in free ZnO- (1µm) in presence of ZnO. E*: Lysinibacillus sp* SEM (1µm) in free ZnO- (1µm) in presence of ZnO**
